# Trauma Hemorrhagic Shock-Induced Lung Injury Involves a Gut-Lymph-Induced TLR4 Pathway in Mice

**DOI:** 10.1371/journal.pone.0014829

**Published:** 2011-08-04

**Authors:** Diego C. Reino, Vadim Pisarenko, David Palange, Danielle Doucet, Robert P. Bonitz, Qi Lu, Iriana Colorado, Sharvil U. Sheth, Benjamin Chandler, Kolenkode B. Kannan, Madhuri Ramanathan, Da Zhong Xu, Edwin A. Deitch, Rena Feinman

**Affiliations:** Department of Surgery, University of Medicine and Dentistry of New Jersey (UMDNJ)- New Jersey Medical School, Newark, New Jersey, United States of America; University of Giessen Lung Center, Germany

## Abstract

**Background:**

Injurious non-microbial factors released from the stressed gut during shocked states contribute to the development of acute lung injury (ALI) and multiple organ dysfunction syndrome (MODS). Since Toll-like receptors (TLR) act as sensors of tissue injury as well as microbial invasion and TLR4 signaling occurs in both sepsis and noninfectious models of ischemia/reperfusion (I/R) injury, we hypothesized that factors in the intestinal mesenteric lymph after trauma hemorrhagic shock (T/HS) mediate gut-induced lung injury via TLR4 activation.

**Methods/Principal Findings:**

The concept that factors in T/HS lymph exiting the gut recreates ALI is evidenced by our findings that the infusion of porcine lymph, collected from animals subjected to global T/HS injury, into naïve wildtype (WT) mice induced lung injury. Using C3H/HeJ mice that harbor a TLR4 mutation, we found that TLR4 activation was necessary for the development of T/HS porcine lymph-induced lung injury as determined by Evan's blue dye (EBD) lung permeability and myeloperoxidase (MPO) levels as well as the induction of the injurious pulmonary iNOS response. TRIF and Myd88 deficiency fully and partially attenuated T/HS lymph-induced increases in lung permeability respectively. Additional studies in TLR2 deficient mice showed that TLR2 activation was not involved in the pathology of T/HS lymph-induced lung injury. Lastly, the lymph samples were devoid of bacteria, endotoxin and bacterial DNA and passage of lymph through an endotoxin removal column did not abrogate the ability of T/HS lymph to cause lung injury in naïve mice.

**Conclusions/Significance:**

Our findings suggest that non-microbial factors in the intestinal mesenteric lymph after T/HS are capable of recreating T/HS-induced lung injury via TLR4 activation.

## Introduction

Acute lung injury (ALI) and the development of the acute respiratory distress syndrome (ARDS) is one of the major causes of death in trauma patients and is associated with mortality rates as high as 40% in intensive care unit patients [1]. The mortality rate of ALI and ARDS has only modestly improved over the last several decades, since the pathophysiology of this syndrome remains incompletely understood and thus therapy remains largely supportive. Consequently, extensive investigative efforts have been focused on clarifying the basic biology of trauma hemorrhagic shock (T/HS)-induced ALI and ARDS. This work has generated several working hypotheses, one of which is that acute lung injury after trauma-hemorrhage is secondary to factors released from the stressed gut [1]. A key element in this gut hypothesis of ARDS is that a splanchnic ischemia-reperfusion (I/R) insult leads to gut inflammation and injury, which in turn results in a gut-induced systemic inflammatory state as well as lung injury. Although gut ischemia has been implicated in the development of ALI, the mechanisms by which a gut I/R event leads to lung injury remains to be fully clarified. Based on our studies showing that T/HS-induced lung injury can be prevented in both rodents and non-human primates by ligating the mesenteric lymph duct [2], [3] and that the injection of T/HS mesenteric lymph, collected from animals subjected to T/HS, into naïve rodents recreates the lung injury observed after actual T/HS [4], we have proposed the hypothesis that gut-derived lymph is the vehicle by which gut injury leads to distant organ injury [5]. The mechanisms by which T/HS lymph is sensed in the lung and the signaling pathways that are involved in T/HS lymph-induced inflammation and injury are not well defined. Since T/HS lymph is sterile and does not contain measurable levels of endotoxin [6], but does contain biologically active non-microbial protein and lipid species [7], [8] it appears that T/HS mesenteric lymph-induced lung injury is being directly mediated by gut-derived endogenous ligands.

Based on studies indicating that the germline-encoded pattern-recognition Toll-like receptors (TLR) recognize molecular motifs shared by large groups of microorganisms [9], [10] as well as by endogenous ligands released from stressed and/or injured tissues [11], [12], we hypothesized that T/HS lymph was exerting its lung injurious effects via a TLR-dependent pathway. In this study, we chose to focus on TLR4 as the putative signaling system by which T/HS lymph induces lung injury for the following reasons. First, TLR4 activation has been implicated in the pathogenesis of organ injury, increased inflammatory gene expression, innate immune system priming and microvascular dysfunction after hemorrhagic shock as well as mechanical and thermal trauma [13], [14], [15], [16], [17], [18], [19], [20] . Secondly, TLRs are constitutively expressed on cells of the innate immune system, including macrophages and neutrophils [9]. Lastly, TLR4 has recently been documented to recognize endogenous danger-type or ‘alarmin’ factors thereby implicating TLR4 as a sensor of tissue injury as well as microbial invasion [21]. Therefore, the goal of this study was to determine whether non-microbial factors in the intestinal mesenteric lymph after T/HS mediate gut-induced lung injury via TLR4 activation.

## Materials and Methods

### Animals

Adult male non-castrated Yorkshire minipigs, weighing 25 to 35 kg were used in this study (Animal Biotech Industries). Male 10–12 week old outbred cesarean-derived (CD-1) mice, TLR4-mutated (C3H/HeJ) mice and their wild-type (WT) littermates (C3H/HeOuJ), TLR-2 deficient (TLR2^−/−^) mice and their WT (C57BL/6J) littermates, and C57BL/6J-Ticam1^Lps2^/J and their WT (C57BL/6J) littermates were obtained from The Jackson Laboratory. Mice on the C57BL/6J background with a specific deletion of the MyD88 gene (MyD88^−/−^ mice) were kindly provided by Dr. Samuel J. Leibovich (UMDNJ-New Jersey Medical School, Newark, N.J). The genotyping of Myd88^−/−^, Myd88^+/−^ and Myd88^+/+^ WT (C57BL/6J) was described previously [22]. C3H/HeJ mice have a point mutation in the TLR4 gene [23] and C57BL/6J-*Ticam1^Lps2^*/J mice have a Lps mutation in the Trif/or Ticam-1 gene that inhibit TLR4- and TRIF-dependent signaling respectively. From herein C3H/HeJ mice will be referred to as TLR4^mut^ and C57BL/6J-*Ticam1^Lps2^*/J mice will be referred to as TRIF^mut^ mice. The animals were maintained in accordance with the guidelines of the National Institutes of Health Guide for Care and Use of Laboratory Animals and the experiments were approved by the Animal Care and Use Committee of the University of Medicine and Dentistry of New Jersey.

### Pig trauma-hemorrhagic shock and lymph collection model

Pigs underwent mesenteric lymph duct cannulation, followed by T/HS or trauma sham-shock (T/SS), as described previously [24]. Briefly, male pigs were subjected to T/HS which involved a laparotomy with mesenteric lymph duct cannulation and withdrawal of blood in a staged fashion to a MAP of 40 mm Hg. The MAP was maintained until the base deficit reached -5 or the total shock period reached 3 hours. Animals were then resuscitated in a staged fashion to a MAP of 80–100 mm Hg. T/SS animals underwent laparotomy with lymph duct cannulation and without blood withdrawal. Mesenteric lymph was collected in sterile tubes for 30 minutes before shock, during shock and on an hourly basis during the postshock period. The collected lymph specimens were centrifuged at 500 g for 20 minutes at 4°C to remove all cellular components, tested for sterility on MacConkey and blood agar plates, aliquoted and stored at −80°C.

### T/HS model

Male mice were anesthetized with pentobarbital (60–80 mg/kg IP) and under strict asepsis, a 2.5-cm midline laparotomy was performed. Isoflurane was given if needed to maintain the surgical level of anesthesia. Blood was withdrawn from the jugular vein until a mean arterial pressure (MAP) between 35–40 mmHg was obtained and maintained for 60 min. After 60 min, the mice were resuscitated with their shed blood. Animals subjected to T/SS underwent cannulation of the femoral artery and jugular vein followed by a laparotomy; however, no blood was withdrawn and the MAP was kept within normal limits. At 3 hr, after the end of shock or sham shock period, the Evans blue dye (EBD) lung permeability assay was performed.

### Lymph infusion protocol

Mice underwent laparotomy as well as internal jugular vein cannulation. Laparotomy was closed after 15 minutes using two layers of 3–0 silk suture. WT, TLR4^mut^, Myd88^−/−^, TRIF^mut^ and TLR2^−/−^ mice were infused with T/HS and T/SS lymph from different pigs. The lymph used for each mouse was collected from an individual pig (pooled fractions collected during 1–3 hr post T/SS or T/HS). T/HS or T/SS pig lymph was infused via the jugular catheter at a rate of 10 µL/g body weight per hour for 3 hours. At the end of the 3 hour lymph infusion period, the mice were euthanized. The EBD lung permeability assay was performed and the lungs were harvested for myeloperoxidase (MPO) levels, and western blotting. The rationale for the lymph volume infused was based on the actual amount of lymph produced by the pigs (ml/kg/hr) subjected to T/HS or T/SS over the shock and resuscitation period which was approximately 28–30 mL/kg [25]. Dose-response pilot studies of pig T/HS lymph documented that lung injury occurred with doses of pig lymph as low as 10 µL/g body weight (data not shown).

### Evans blue dye (EBD) lung permeability assay

Mice were injected with EBD (30 mg/kg in 4% bovine serum albumin) via the internal jugular catheter 30 min prior to the end of the 3 hr reperfusion period following T/HS or T/SS or 30 min prior to the end of the 3 hr lymph infusion. After 10 minutes (allowing complete circulation of the dye), a blood sample (0.3 mL) was withdrawn from the femoral artery catheter, centrifuged and the resultant plasma was serially diluted to form a standard curve for EBD. After 20 min of EBD injection, a cardiopulmonectomy was performed after the mouse was fully exsanguinated. A bronchoalveolar lavage was then performed on the excised lungs. 1 ml of phosphate buffered saline (PBS) was instilled, rinsed in and out 3 times, repeated twice and the bronchoalveolar lavage fluid (BALF) was collected, centrifuged and the absorbance of EBD (620 nm) in the supernatant was measured spectrophotometrically. The percentage of EBD in the BALF was determined relative to the standard curve of EBD in the plasma.

### Myeloperoxidase assay

Frozen lung tissue was homogenized and assayed for MPO activity as previously described [2]. One unit of MPO activity represents the amount of enzyme that will reduce 1 mol/min of peroxide.

### Western blot analysis

Whole cell extracts (WCE) prepared from lung tissue as previously described [26] were resolved by SDS-PAGE and detected by western blotting. The following antibodies were used: rabbit anti-iNOS (1∶3000; BD Biosciences) and rabbit anti-p42/p44 (1∶1000, Cell Signaling). As reported by others [27], total p42/p44 was used as our loading control since the expression of commonly used loading controls such as actin and tubulin have been shown to be altered in tissues of animals subjected to I/R injury [28]. The western blots were developed with immoblin western (Millipore) and then analyzed by densitometry using an AlphaImager 3400 imaging system and AlphaEase FC software (Alpha Innotech).

### Measurement of endotoxin in mesenteric lymph and endotoxin removal process

Lymph pooled from different pigs (n = 4–5) collected from the first 3 hr after T/HS were used in these assays. Endotoxin levels in the mesenteric lymph samples were measured using the limulus lysate assay pyrogent plus test kit according to the instructions provided by Lonza. Each sample was heat treated at 70°C for 5 minutes to inactivate potential inhibitory factors present in the mesenteric lymph and run in duplicate. The limit of detection was 0.06 endotoxin units (EU) /ml.

To further exclude the possibility that endotoxin is responsible for the lung injurious effects of the T/HS lymph samples, endotoxin removing columns containing immobilized polymyxin B that binds and removes endotoxin (Detoxi-Gel Endotoxin Removing Columns, Thermo Scientific Pierce) were used according to the manufacturer's instructions. To validate the efficacy of the columns, PBS and lymph specimens were spiked with 0.25 EU of endotoxin (*E. coli* 055:B5, Lonza) and endotoxin levels of spiked samples were measured using the limulus assay both prior to being placed on the column as well as after passage through the columns. Pre- and post-polymyxin-immobilized column eluted T/SS and T/HS lymph samples were infused in naïve C57BL6 mice for 3 hours as described above.

### Isolation and Detection of Bacterial DNA in Mesenteric Porcine Lymph

DNA was isolated from 500 µL of porcine mesenteric lymph. The samples were centrifuged at 20,000xg for 10 min at 4°C and the pellet was lysed using MagNA Pure bacteria lysis buffer according to the manufacturer's instructions (Roche Diagnostics GmBH). DNA was isolated using the QIAamp DNA Mini Kit (Qiagen). DNA was also isolated from *E. Coli* as a positive control for bacterial DNA. PCR reactions for the amplication of the 16S ribosomal RNA wereperformed. The 16S rRNA primers were synthesized in the Molecular Resource Facility (New Jersey Medical School). The sequences of the 16S primer set included the forward primer (5′-TCCTACGGGAGGCAGCAGT-3′) and reverse primer (5′-GGACTACCAGGGTATCTAATCCTGTT-3′) as described in [29]. The PCR reaction was performed with 5 ng of template and 50 pM of each primer using Platinum Taq polymerase (Invitrogen) in a thermal cycler 2720 (Applied Biosystems). DNA from E.coli served as a positive control and sterile water without template served as a negative control. Thermocycling conditions were: 95°C for 4 min, 25 cycles at 94°C for 30 s, 58°C for 30 s and 72°C for 30 s followed by a final extension at 72°C for 7 min. A 466 bp PCR 16S rRNA product was visualized on an 1.5% agarose gel stained with ethidium bromide.

### Statistics

Analysis of variance (ANOVA) with the post hoc Tukey-Kramer multiple comparison test was used for comparisons between groups. Results are expressed as mean ± SE. *P* values less than 0.05 were considered statistically significant.

## Results

### Infusion of porcine mesenteric T/HS lymph induces lung injury in naïve CD1 mice

**Figure 1:**
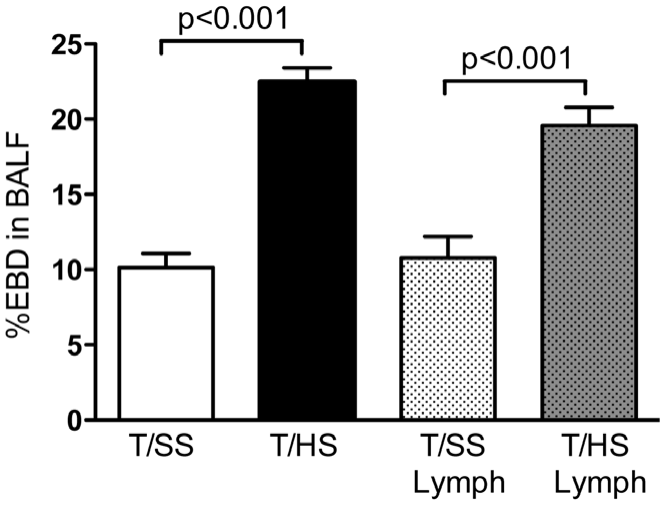
Pig T/HS lymph infusion in naïve CD1 mice recreates T/HS-induced lung injury. Lung permeability to Evans Blue dye (EBD) was measured in CD-1 mice subjected to actual T/HS or T/SS for 60 min and 3 hr reperfusion as well as CD1 mice infused with porcine T/SS or T/HS lymph for 3 hr. Data expressed as mean ± SE (n = 5–6 mice/ group).

Our earlier studies demonstrated that the infusion of rat T/HS mesenteric lymph into naïve mice recreated the lung injury observed with actual T/HS [4]. As reported in our *in vitro* studies using T/HS lymph from rats [2], pigs [25] and baboons [3] mesenteric lymph collected from male pigs subjected to T/HS contributed to priming of neutrophils and endothelial dysfunction. Thus, we first determined whether the infusion of sterile porcine mesenteric T/HS lymph into naïve CD1 mice caused similar lung injury as observed in CD1 mice subjected to T/HS. The rationale for using CD1 mice is that these mice have a robust immune system and do not have any of the potential confounding factors associated with inbreeding. As shown in Fig. 1, the infusion of pig T/HS lymph increased lung permeability to EBD by 2-fold as compared to CD-1 mice infused with T/SS pig lymph. Likewise, a 2.3-fold increase in percentage of EBD in the BALF was found in CD-1 mice subjected to actual T/HS as compared to their T/SS counterparts.

### TLR4 deficiency attenuates porcine T/HS lymph-induced lung injury

**Figure 2:**
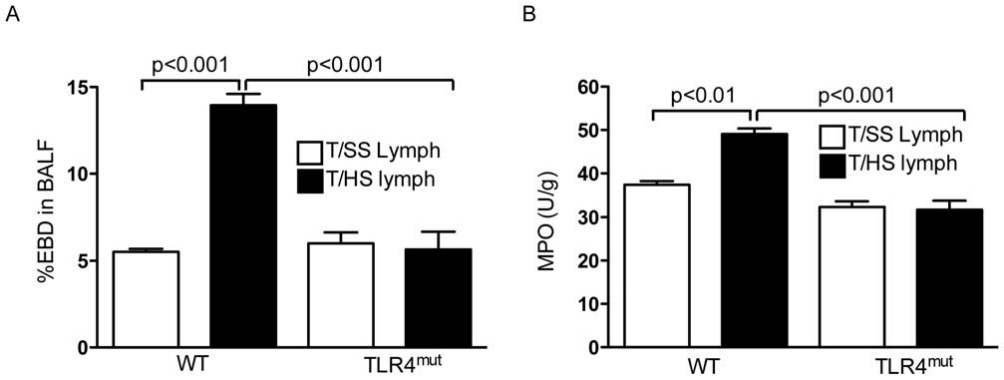
TLR4 deficiency attenuates T/HS lymph infused lung injury. WT and TLR4^mut^ mice were infused with porcine T/SS and T/HS lymph for 3 hr. A) Lung permeability to EBD was performed. Data expressed as mean ± SE (n = 3–12 mice/ group). B) MPO levels (U/g) were measured in lung homogenates. Data expressed as mean ± SE (n = 4–7 mice/group).

Having documented that T/HS lymph can recreate lung injury, we tested the hypothesis that non-microbial factors in the intestinal mesenteric lymph after T/HS mediate gut-induced lung injury via TLR4 activation. WT and TLR4^mut^ mice were infused with porcine T/SS or T/HS lymph for 3 hours. Infusion of WT mice with T/HS lymph increased lung permeability by 2.5-fold as compared to mice infused with T/SS lymph (Fig. 2A). In contrast, there was no significant increase in lung permeability in TLR4 deficient mice infused with T/HS lymph relative to their T/SS lymph infused counterparts. Since the sequestration of polymorphonuclear neutrophils (PMNs) into the lung microvasculature has been characterized as a hallmark of ALI, MPO levels were measured in lung homogenates. As shown in Fig. 2B, a modest increase in MPO activity was evident in WT mice infused with T/HS lymph compared to WT mice infused with T/SS lymph. There was no difference between MPO levels in TLR4^mut^ mice infused with T/HS or T/SS lymph. These findings suggest that factors in T/HS lymph mediate lung injury via TLR4 activation.

**Figure 3:**
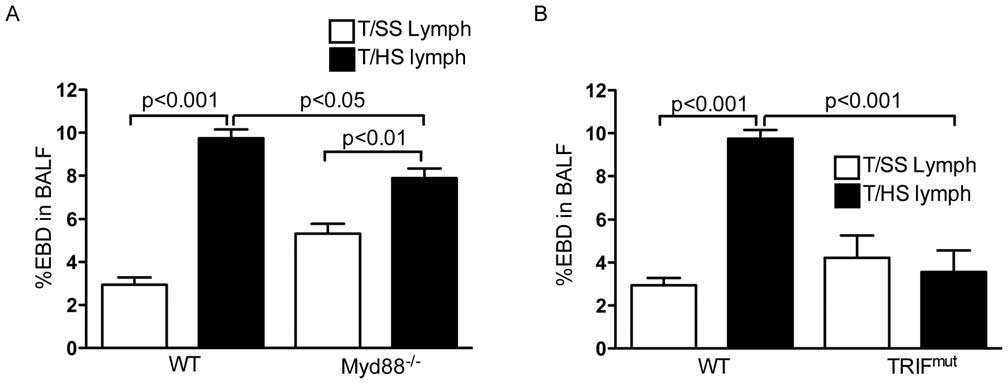
TRIF and Myd88 deficiency confer full and partial protection against T/HS lymph induced microvascular permeability. A) WT and Myd88^−/−^ and B) WT and TRIF^mut^ mice were infused with porcine T/SS and T/HS lymph for 3 hr and lung permeability to EBD was performed. Data expressed as mean ± SE (n = 5–8 mice/ group).

Since TLR4 mediates its downstream effects via two distinct signaling pathways, Myd88 and TRIF, we determined the effects of Myd88 and TRIF deficiency on T/HS lymph-induced lung injury. In both WT and Myd88^−/−^ mice, infusion with T/HS lymph increased lung permeability by 3.3-fold (p<0.001) and 1.5-fold (p<0.01) respectively versus their T/SS lymph infused counterparts (Fig. 3A). The baseline level of lung permeability for the T/SS lymph infused Myd88^−/−^ group was approximately 1.8-fold higher than the WT T/SS lymph infused group (p<0.05). In contrast, there was no significant increase in the percentage of EBD in the BALF of TRIF^mut^ mice infused with T/HS lymph relative to their T/SS lymph infused group (Fig. 3B). Although both TRIF and Myd88 deficiency attenuated T/HS lymph-induced lung permeability (p<0.001 and p<0.05 respectively vs WT T/HS lymph infused mice), our results suggest that TRIF plays a more predominant role in T/HS lymph-induced lung permeability.

**Figure 4:**
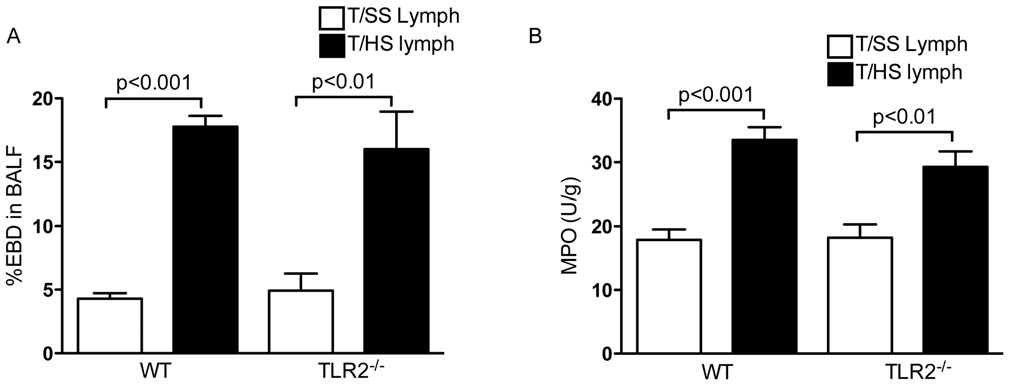
TLR2 does not mediate T/HS lymph-induced lung injury. WT and TLR2^−/−^ mice infused with porcine T/HS and T/SS lymph for 3 hr. A) Lung permeability to EBD was performed. Data expressed as mean ± SE (n = 4–6 mice/ group). B) MPO levels (U/g) were measured in lung homogenates. Data expressed as mean ± SE (n = 5–6 mice/group).

To further confirm the specificity of the TLR4 response in T/HS lymph-induced lung injury, we examined the effects of TLR2 deficiency on lung injury. In both the WT and TLR2 deficient mice, infusion with T/HS lymph increased lung permeability and pulmonary leukosequestration, as determined by EBD permeability (Fig. 4A) and MPO levels (Fig. 4B).

### TLR4 deficiency blunts the pulmonary iNOS response associated with T/HS lymph-induced lung injury

**Figure 5:**
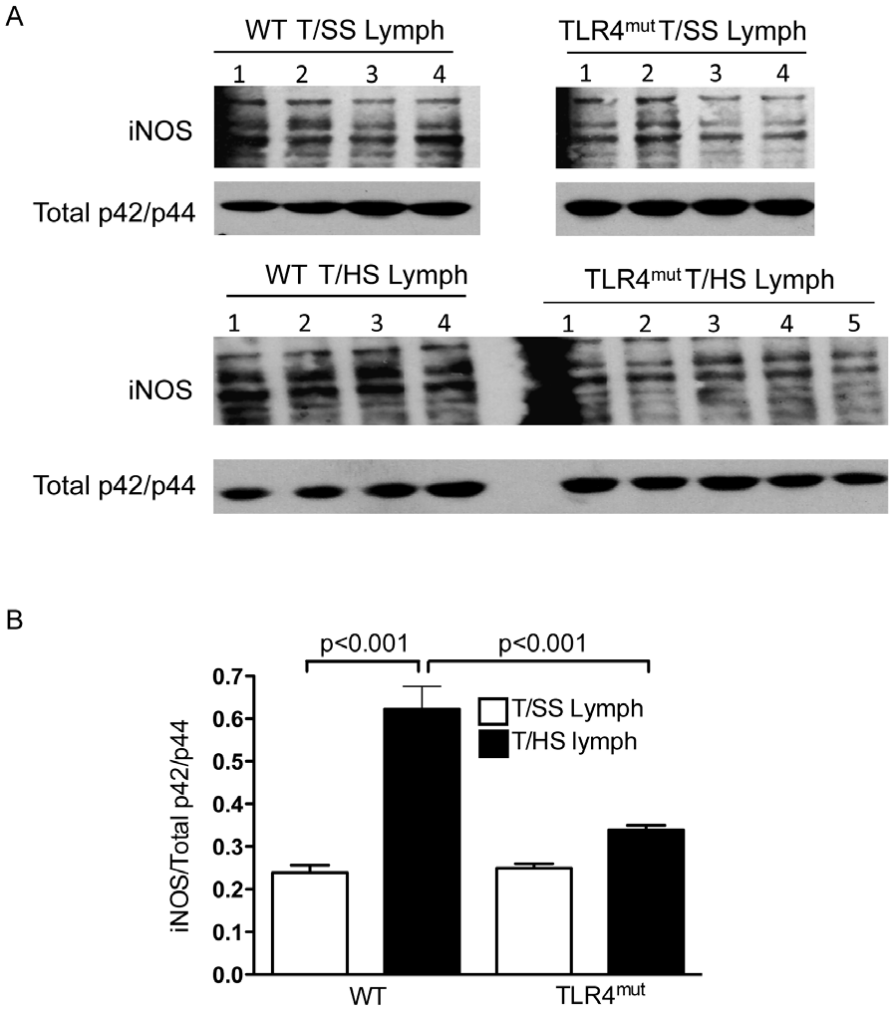
TLR4 deficiency reduces T/HS lymph induced pulmonary iNOS protein levels. A) and B) Western blot of iNOS in lung WCEs of WT and TLR4^mut^ mice infused with porcine T/SS and T/HS lymph for 3 hr. B) Densitometry was performed to quantify iNOS and total p42/p44 MAPK expression. Data expressed as mean ± SE (n = 4–7 mice/group).

Since increased iNOS activity has been implicated in T/HS-induced lung injury [4] and iNOS is a downstream target of TLR4 activation [21], we examined the effects of TLR4 deficiency on the pulmonary iNOS response after T/HS lymph infusion. Compared with WT mice infused with T/SS lymph, iNOS protein levels were 3-fold higher in WT mice infused with T/HS lymph (Fig. 5A and B). The induction of iNOS expression in lung homogenates from TLR4^mut^ mice infused with T/HS lymph was markedly reduced as compared to their WT counterparts but remained elevated as compared to TLR4^mut^ mice infused with T/SS lymph. Lungs from WT and TLR4^mut^ mice infused with T/SS lymph expressed negligible levels of iNOS protein. These findings suggest an association between the T/HS lymph-induced pulmonary iNOS response and TLR4 activation.

### Endotoxin-and bacterial DNA- independent factors in T/HS lymph mediate lung injury

**Figure 6:**
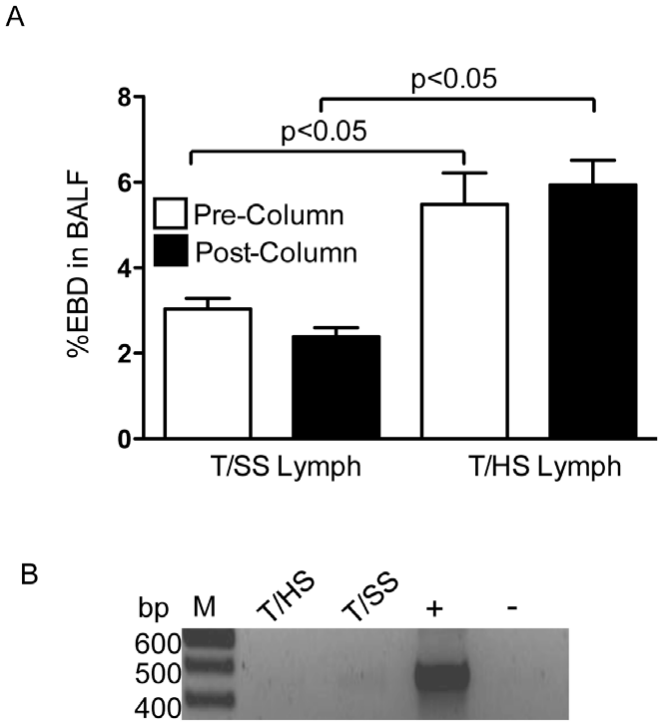
Endotoxin- and bacterial DNA-independent factors in T/HS lymph mediate lung injury. A) Pre- and post-polymyxin-immobilized column eluted T/SS and T/HS lymph samples were infused in naïve C57BL6 mice for 3 hours. Lung permeability to EBD. Data expressed as mean ± SE (n = 3–9 mice/ group). B) DNA isolated from lymph pooled from 3 pigs subjected to either T/HS or T/SS lymph was tested for bacterial DNA contaminants by PCR using 16S rDNA primers. DNA isolated from *E.Coli* served as our positive control was depicted as + and the negative control containing no DNA template was depicted as -. M denotes a 100 bp size marker.

Although the lymph samples tested had no bacterial growth and were sterile (data not shown), we needed to rule out the possibility that the activation of TLR4 by T/HS lymph was due to gut-derived endotoxin contamination. Using a limulus lysate assay, neither T/SS nor T/HS lymph samples contained detectable levels of endotoxin by the limulus lysate assay (level of detection 0.06 EU/ml). To exclude the possibility that inhibitory factors in the lymph samples were interfering with the limulus assay, known quantities of endotoxin (0.06 EU, 0.125 EU or 0.25 EU) were added to T/SS and T/HS lymph samples and then assayed for endotoxin levels. The limulus assay tested positive for all the LPS concentrations tested, including 0.06 EU/ml of endotoxin, thereby validating that the limulus assay was able to detect endotoxin present in the lymph samples (data not shown). To further confirm that factors in T/HS lymph that mediate lung injury and activate TLR4 are devoid of endotoxin, pre- and post-polymyxin-immobilized column eluted T/SS and T/HS lymph samples were infused in naïve C57BL6 mice for 3 hours. As shown in Figure 6A, the magnitude of lung permeability was similar in mice infused with pre- and post-column T/HS lymph samples as compared to mice infused with T/SS lymph samples. Additionally, PCR analysis was performed to detect bacterial DNA in T/SS and T/HS lymph using a primer set which specifically detects 16S rDNA of the Domain *Bacteria* [29] and neither T/HS nor T/SS lymph tested positive for bacterial DNA contamination (Fig. 6B).

## Discussion

Studies investigating the pathophysiology of trauma-induced SIRS, ARDS and multiple organ dysfunction syndrome (MODS) have been a major area of investigation. Work by us [1], [2], [3], [30] and others [31], [32], [33], [34], [35] have demonstrated that splanchnic ischemia leading to gut inflammation and loss of barrier function is the initial triggering event that turns the gut into the ‘motor of MODS’. Although, it was originally proposed that bacterial (endotoxin) translocation was the link between gut and distant organ injury, most recently, using rodent [36], porcine [24], and non-human primate trauma-hemorrhagic shock [3] as well as burn models [37], we have documented that acute ARDS and MODS observed after a major injury is due to immuno-inflammatory, tissue-injurious factors exiting the gut via the intestinal lymphatics. More support for the gut-lymph hypothesis of MODS stems from our recent study demonstrating that naïve rats and mice infused with mesenteric lymph from rats subjected to T/HS develop ALI [4]. Our current study extends these results by showing that T/HS lymph-induced lung injury occurs via a TLR4-dependent pathway. The notion that TLR4, as well as other pattern recognition receptors, can respond to non-bacterial endogenous danger-type molecules as well as microbial products has expanded the role of pattern recognition receptors beyond that of sensors of microbial invasion [38]. This concept also helps explain the many similarities between the host's septic response and the augmented inflammatory response to shock-trauma and other forms of major tissue injury.

Our results implicating TLR4, but not TLR2, as the proximal receptor for T/HS lymph-induced lung injury is novel in that we have implicated non-bacterial factors in mesenteric lymph that are able to directly activate TLR4. These findings are consistent with other studies showing that hemorrhagic shock-induced lung injury [16], and liver injury [13], [18] is reduced in TLR4 deficient mice but not TLR2 deficient mice. Since other non-infectious models of injury, such as femur fracture [18], [39] and thermal injury [20] also document that TLR4 deficient animals are protected, it appears that this immune-activating and tissue-injurious effect of TLR4 may be a common response to diverse shock and tissue-injury states. On the other hand, in certain conditions, such as hyperoxia-induced lung injury, TLR4 activation has been shown to be protective [40], [41]. Thus, like many other systems, whether TLR4 activation is protective or deleterious may depend on the condition being studied as well as the timing and magnitude of its activation. Nonetheless, it is now clear that during tissue injury or proteolysis, extracellular matrix components undergo cleavage, exposing moieties that can act as ligands for TLRs and thereby initiate TLR-induced signal transduction. In this way, innate immune inflammatory responses may be activated without the presence of invading bacteria, but rather as a direct consequence of tissue injury [21].

Depending on the type, magnitude and duration of insult as well as the site of injury, the specificity of the TLR4 response is determined by the selective recruitment of its adapters, namely Myd88/TIRAP and TRIF/TRAM and their respective downstream signaling. Although there have been extensive studies delineating the Myd88-dependent and TRIF-dependent intracellular signaling pathways in the pulmonary innate immune response to microbial infections, our knowledge of their downstream signaling cascades in models of sterile/noninfectious ALI is very limited. For example, HMGB1/TLR4/Myd88 signaling activates NAD(P)H oxidase in neutrophils after hemorrhagic shock [42] whereas oxidized phospholipids mediate acid-induced ALI via TRIF signaling [43]. Our study shows full and partial protection against T/HS lymph induced microvascular damage in TRIF and Myd88 deficient mice respectively. However, more work must be done to determine whether TRIF activation plays a more predominant role in the lung injurious effects of T/HS lymph than Myd88 activation.

Among the cells that bear TLR4 are those of the innate immune system, including macrophages, neutrophils and dendritic cells [9]. Since TLR4 activated inflammatory cells manifest increased nitric oxide production [44], [45], we asked whether TLR4 activation in T/HS lymph-induced lung injury was associated with the induction of the iNOS response. Our findings demonstrated that lung iNOS expression was markedly but not completely reduced in TLR4^mut^ mice infused with T/HS lymph as compared to iNOS expression in WT mice. Although this observation suggests that the induction of the iNOS response by T/HS lymph is modulated by both TLR4-dependent and TLR4-independent pathways, the concept that up-regulated iNOS activity is involved in the pathogenesis of T/HS-induced lung injury is consistent with studies showing that excessive nitric oxide production contributes to trauma-shock-induced organ injury [46], [47], [48], [49]. Thus, taken together, our previous work showing iNOS deficient mice are resistant to T/HS lymph-induced lung injury [4] and the current study documenting that TLR4 deficient mice are also resistant to T/HS lymph-induced lung injury provide important clues as to how T/HS is converted from a hemodynamic insult into an inflammatory and lung tissue injurious disease.

Having demonstrated that T/HS lymph is capable of recreating shock-induced lung injury through TLR4- and iNOS-dependent pathways, the question arises about the composition of T/HS lymph and what factors within it are triggering this response. Although T/HS lymph is collected from animals subjected to T/HS, in which gut barrier function is impaired, we do not believe that intestinal bacteria and/or endotoxin are responsible for the biologic activity of T/HS lymph. This conclusion is based on our current results showing that the pig lymph samples were devoid of bacteria, bacterial DNA and did not contain measurable levels of endotoxin. Furthermore, the biologic lung-injurious activity was not abrogated after passage through an endotoxin removal column. The notion that injury-induced TLR4 activation is not due to endotoxin is also supported by studies showing that TLR4 deficient but not CD14 deficient mice were resistant to liver injury after major trauma [18]. Additionally, if gut-derived bacterial products in T/HS lymph are involved in lymph-induced lung injury, gram-positive (peptidoglycan) as well as gram-negative (endotoxin) bacterial products would be present in T/HS lymph. Since peptidoglycan activates TLR2 and TLR2^−/−^ mice are not resistant to T/HS lymph-induced lung injury, it is not likely that gram-positive microbial factors were responsible for the lung-injurious effects of T/HS lymph. The fact that TLR4 has the capacity to recognize and respond to endogenous ligands is also consistent with recent observations showing that T/HS contains several incompletely identified and biologically-active non-cytokine protein and lipid factors [6], [7], [8] including a modified albumin species [7]. Thus, we believe that T/HS lymph contains modified proteins and lipids which could either act as endogenous TLR4 ligands or promote the release of danger signals from a stressed or activated cell population within the lung. Regardless of whether factors in the mesenteric T/HS lymph are acting directly or indirectly in the induction of the TLR4 response, our findings demonstrate that these factors play a role in the sterile inflammatory process that occurs after trauma-hemorrhage.

One advantage of the T/HS lymph infusion model is that it provides a unique opportunity to study the effects of shock-induced, gut-derived factors *in vivo* without the confounding systemic effects of trauma and hemorrhagic shock. Since T/HS lymph is produced *in vivo* and the amounts of lymph required to cause lung injury were equal to or less than what would be produced during the actual trauma-shock period, speaks to the physiologic relevance of the model. Furthermore, since both rat and pig T/HS lymph cause lung injury in mice, a cross-species event, it appears that the factor or factors within T/HS lymph may be conserved among different species and likely target the same pattern recognition receptors. This cross species observation is also important since pigs are physiologically closer to humans than rodents. Taken together, these results lend further support for the Danger model of immune activation, which states that our immune system is not only activated by foreign pathogens, but also by our own endogenous ligands, whose patterns are evolutionarily preserved [50].

In summary, our studies suggest that TLR4 activation by T/HS lymph is necessary for T/HS lymph-induced lung injury and an augmented pulmonary iNOS response. More importantly, our results suggest that non-bacterial factors in T/HS mesenteric lymph mediate cross talk between tissue injury in the gut and subsequent gut-induced lung injury. Future studies elucidating the contribution of TLR4 and its downstream signaling in both immune and parenchymal cell populations that mediate T/HS lymph-induced lung injury as well as identifying endogenous TLR4 ligands after T/HS will provide more insight in the pathogenesis of shock-induced ALI and identify potential therapeutic targets.
